# Involvement of Hepatic SHIP2 and PI3K/Akt Signalling in the Regulation of Plasma Insulin by Xiaoyaosan in Chronic Immobilization-Stressed Rats

**DOI:** 10.3390/molecules24030480

**Published:** 2019-01-29

**Authors:** Qiuxia Pan, Jiajia Wu, Yueyun Liu, Xiaojuan Li, Jiaxu Chen

**Affiliations:** 1 School of Basic Medical Science, Beijing University of Chinese Medicine, Beijing 100029, China; pqx1126@sina.com (Q.P.); wujiajia92@hotmail.com (J.W.); chloelou@126.com (Y.L.); 2 Institute of Basic Theory for Chinese Medicine, China Academy of Chinese Medicine Science, Beijing 100700, China; 3 Formula-pattern Research Center, School of Traditional Chinese Medicine, Jinan University, Guangzhou 510632, China

**Keywords:** chronic stress, Chinese medicinal formulae, metabolic disorders, insulin, lipid

## Abstract

Background: Long-term exposure to chronic stress is thought to be a factor closely correlated with the development of metabolic disorders, such as diabetes mellitus and metabolic syndrome. Xiaoyaosan, a Chinese herbal formula, has been described in many previous studies to exert anxiolytic-like or antidepressant effects in chronically stressed rats. However, few studies have observed the effects of Xiaoyaosan on the metabolic disorders induced by chronic stress. Objective: We sought to investigate the effective regulation of Xiaoyaosan on 21-day chronic immobility stress (CIS, which is 3 h of restraint immobilization every day)-induced behavioural performance and metabolic responses and to further explore whether the effects of Xiaoyaosan were related to SHIP2 expression in the liver. Methods: Sixty male Sprague Dawley rats were randomly divided into a control group, a CIS group, a Xiaoyaosan group and a rosiglitazone group. The latter three groups were subjected to 21 days of CIS to generate the stress model. After 21 days of CIS, the effects of Xiaoyaosan on body weight, food intake, and behaviour in the open field test, the sucrose preference test and the forced swimming test were observed following chronic stress. Plasma insulin, cholesterol (CHOL), triglyceride (TG), low-density lipoprotein (LDL-C) and high-density lipoprotein (HDL-C) concentrations and blood glucose were examined, and the protein and mRNA expression levels of SHIP2, p85 and Akt in the liver were measured using RT-qPCR and immunohistochemical staining. Results: Rats exposed to CIS exhibited depression-like behaviours, decreased levels of plasma insulin, CHOL, LDL-C, TG and HDL-C, and increased blood glucose. Increased SHIP2 expression and reduced Akt, p-Akt and p85 expression were also observed in the liver. Xiaoyaosan exerted antidepressant effects and effectively reversed the changes caused by CIS. Conclusions: These results suggest that Xiaoyaosan attenuates depression-like behaviours and ameliorates stress-induced abnormal levels of insulin, blood glucose, CHOL, LDL-C and HDL-C in the plasma of stressed rats, which may be associated with the regulation of SHIP2 expression to enhance PI3K/Akt signalling activity in the liver.

## 1. Introduction

Epidemiological data have suggested that there are strong associations between chronic stress exposure and metabolic disease [1]. As shown in several studies, prolonged exposure to chronic stress can manifest as stress-related disorders, including metabolic disorders such as diabetes mellitus and metabolic syndrome [2,3,4]. Stress stimulates the hypothalamic–pituitary–adrenal (HPA) system, resulting in increased corticosteroid release, which, along with other stress hormones, affects various aspects of metabolism and physiology [5]. For example, acute restraint stress for 2 h [6], acute swimming stress [7], social protest stress [8] and chronic immobilization stress [9] caused significant increases in plasma corticosterone levels in male rats. In addition, foot-shock stress and psychological stress increased the concentration of corticosterone, glucose, insulin, cholesterol and triglycerides in the plasma of male rats [10]. Understanding the effects of stress on various systems is of great significance for maintaining mental and physical health.

The liver is a predominantly metabolic organ that plays diverse biological roles and impacts other systems of the body [11]. It plays a major role in blood glucose homeostasis by maintaining a balance between the uptake and storage of glucose through glycogenesis and the release of glucose via glycogenolysis and gluconeogenesis [12]. In addition, the liver is the principal site of lipid synthesis and storage and is involved in energy balance and lipid homeostasis [13]. Chronic stress can stimulate hepatic gluconeogenesis, inhibit glucose uptake by peripheral tissues, mobilize fat storage as an energy substrate for the liver and suppress immune responses [5]. Although the exact mechanism of metabolic and physiological changes triggered by stress stimulation remains to be clarified, stress-induced metabolic and physiological changes may be related to the altered pathology and physiology of the liver.

The PI3K/Akt signalling pathway, as the main signalling pathway of insulin, participates in the pathophysiological mechanism of glycolipid metabolism in vivo [14]. Phosphatidyl inositol 3-kinase (PI3K) is a class of enzymes that specifically catalyses phosphatidyl inositol phosphorylation of one or more phosphatidylinositol triads on the inositol ring. It is a heterodimer composed of a regulatory subunit, p85, and a catalytic subunit, p110 [15,16,17], the regulatory subunits contain SH2 and SH3 domains and interact with target proteins containing corresponding binding sites. Akt is a serine/threonine protein kinase, also known as protein kinase B (PKB), which plays a role in cell growth, survival, metabolism, carcinogenic transformation and other biological processes, and Akt is the main signal transducer downstream of PI3K in various signal cascades [18,19]. Recently, subtypes of Akt, i.e., Akt1, Akt2 and Akt3, were identified, and these subtypes have high sequence homology, among which Akt1 and Akt2 are significantly expressed in classic insulin-sensitive tissues such as liver, muscle and fat [20].

SHIP2 is a member of the class II inositol 5-phosphatase family, which is ubiquitously expressed in mammalian cells [21]. SHIP2 is a lipid phosphatase that inhibits insulin signalling downstream of phosphatidylinositol 3-kinase (PI3K) [22]. SHIP2 containing SH2 domain can bind to p85 and inhibit downward transmission of PI3K/Akt signalling. Additionally, SHIP2 is required to maintain normal systemic glucose homeostasis and prevent oxidative stress-induced endothelial dysfunction [23]. Studies using SHIP2 gene-deficient mice have provided evidence that SHIP2 is a powerful negative regulator of insulin signalling and insulin sensitivity in vivo and plays an important role in improving insulin sensitivity and regulating lipid metabolism [24,25].

Rosiglitazone, a peroxisome proliferator-activated receptor gamma (PPARγ) agonist, was approved as a conventional insulin sensitizer in the treatment of type 2 diabetes mellitus [26]. Rosiglitazone reduces plasma glucose concentration and glucose production and increases glucose clearance in patients with type 2 diabetes [27,28], and several studies have shown that rosiglitazone has a certain effect on the PI3K/Akt signalling pathway [29,30,31]. In addition to being used as a treatment for diabetes mellitus, rosiglitazone has been found to exert neuroprotective effects [32]. Therefore, rosiglitazone was used as a control drug in this study to explore the effect of Xiaoyaosan on the glycolipid metabolism of CIS-induced rats.

Our previous studies have indicated that Xiaoyaosan has a regulatory effect on mental behaviour disorders caused by chronic immobilization stress (CIS). For example, Xiaoyaosan effectively regulated the expression levels of brain-derived neurotrophic factor (BDNF) and its receptors (TrkB) in the hippocampus, thereby helping to reduce CIS-induced depression [33]. Xiaoyaosan also suppressed the hyperactivation of the HPA axis to ameliorate stress-related depression-like behaviours [34]. Moreover, Xiaoyaosan down-regulated the activity of the TNF-alpha/JAK2–STAT3 signalling pathway in the hippocampus to exert stress-induced anxiolytic-like effects [35]. However, few studies have explored the effects and potential mechanisms of Xiaoyaosan on stress-induced metabolic disorders. Therefore, the purpose of the present study was to investigate the effects of chronic stress on glycolipid metabolism and the regulatory effect of Xiaoyaosan. We preliminarily evaluated the changes in body weight, food intake, plasma insulin, blood lipid and blood glucose in CIS-induced rats after Xiaoyaosan treatment. Additionally, to further explore the mechanism of this regulatory effect, the protein and gene expression levels of SHIP2, p85 and Akt in the liver were measured.

## 2. Result

### 2.1. Effects of Xiaoyaosan on Weight and Food Intake in Rats Exposed to CIS

**Figure 1:**
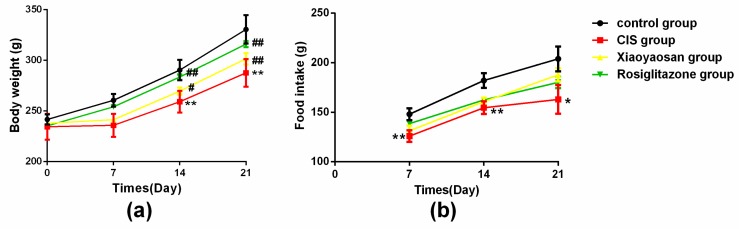
Effect of Xiaoyaosan on stress-induced changes in weight and food intake. The body weight and food intake of rats were recorded during the 21-day CIS period. (**a**) Changes in weekly body weight of each group of rats. (**b**) Changes in weekly food intake of each group of rats. Data are presented as the means ± SEM with seven rats in each group. * *p* < 0.05 or ** *p* < 0.001 versus the control group. ^#^ *p* < 0.05 or ^##^ *p* < 0.001 versus the CIS group.

The CIS rat model was established in the present study to verify whether the CIS protocol affected body weight and basic daily intake in rats. Body weight and food intake of each rat was monitored before initiating the CIS regimen and then weekly or daily until the end of the CIS procedure. The rats in the CIS group had a significantly lower body weight in the 2nd and 3rd weeks than those in the control group (Figure 1a, *p* < 0.001), and the body weight of the CIS group increased slowly. Xiaoyaosan or rosiglitazone treatment significantly increased body weight compared with the CIS group in the 2nd week (*p* < 0.05 and *p* < 0.001, respectively) and 3rd week (both *p* < 0.001). The amount of weekly food intake significantly differed between the control and CIS groups in the 1st and 2nd weeks (Figure 1b, *p* < 0.001 and *p* < 0.05). Xiaoyaosan treatment did not significantly increase the weekly food intake compared with the CIS group. Rosiglitazone treatment significantly increased the weekly food intake compared with the CIS group (*p* < 0.05) in the 1st week, but there was no significant difference between the two groups in the 2nd and 3rd weeks, demonstrating that the effect of Xiaoyaosan on the body weight of CIS rats was not due to increased food intake.

### 2.2. Effects of Xiaoyaosan on Behavioural Changes in Rats Exposed to CIS

We conducted a series of behavioural tests, including the open field test (OFT), forced swimming test (FST) and sucrose preference test (SPT), to examine the effects of Xiaoyaosan on behavioural changes in rats exposed to CIS.

**Figure 2:**
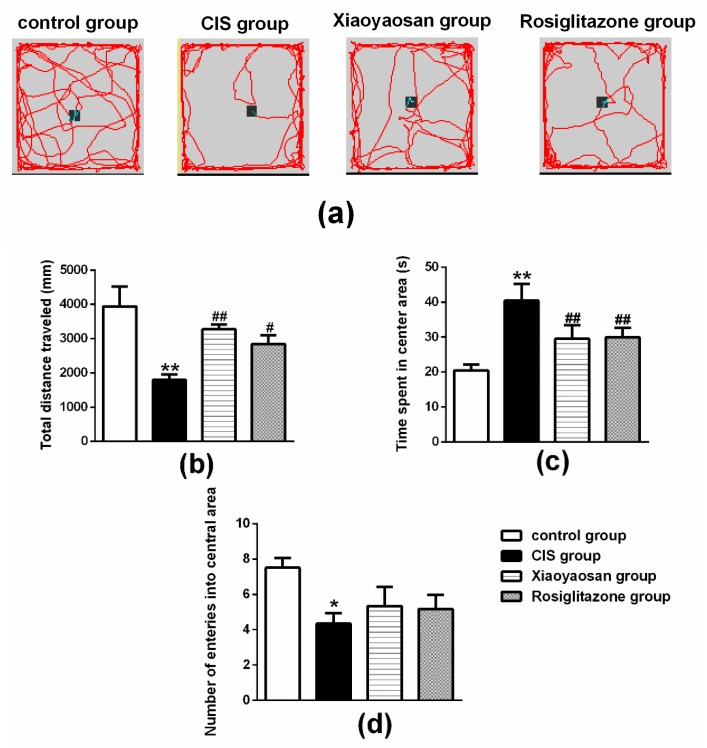
Effects of Xiaoyaosan on stress-induced locomotor activity and exploratory behaviour. (**a**) The track maps of the rats in the different groups in the OFT test. (**b**) Total distance travelled in the OFT. (**c**) The time spent in the centre area of the OFT. (**d**) Number of times the centre area was entered in the OFT. Data are presented as the means ± SEM with 9–10 rats in each group. * *p* < 0.05 or ** *p* < 0.01 versus the control group. ^#^ *p* < 0.05 or ^##^ *p* < 0.01 versus the CIS group.

For the results of the OFT shown in Figure 2b, there was a significant decrease in the total distance travelled in the OFT in the CIS group (*p* < 0.01), while the total distance travelled in the OFT of CIS-induced rats significantly increased after administration of Xiaoyaosan or rosiglitazone (*p* < 0.001 and *p* < 0.05, respectively). As shown in Figure 2c, the CIS-exposed rats spent significantly more time in the centre area than the control group (*p* < 0.01). Both the Xiaoyaosan and rosiglitazone treatments remarkably reversed the CIS-induced increase in the time spent in the open area (*p* < 0.01). Similarly, a significant reduction in the number of times the centre area was entered (Figure 2d) was observed in the CIS groups compared with the control group (*p* < 0.05); neither Xiaoyaosan nor rosiglitazone ameliorated this reduction (*p* > 0.05).

**Figure 3:**
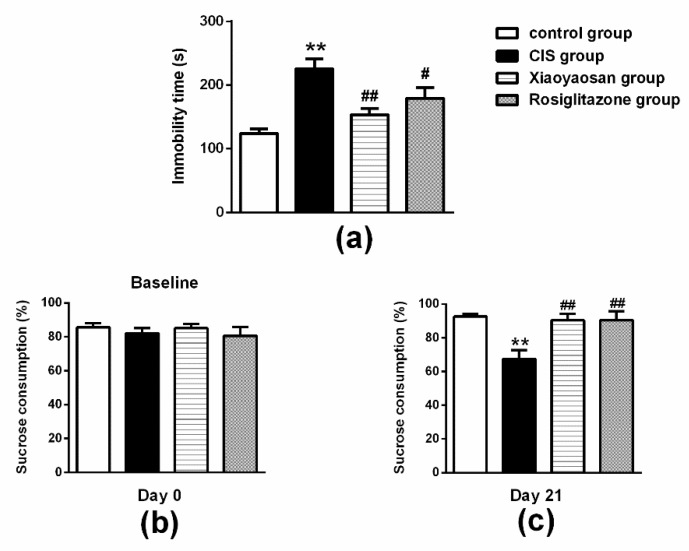
Effects of Xiaoyaosan on stress-induced depression-like behaviour. (**a**) Immobility time within 5 min in the FST. (**b**) Sucrose consumption rate in the SPT before CIS. (**c**) Sucrose consumption rate in the SPT after 21 days of CIS. Data are presented as the means ± SEM with 9–10 rats in each group. ** *p* < 0.01 versus the control group. ^#^ *p* < 0.05 or ^##^ *p* < 0.01 versus the CIS group.

For the results of the FST shown in Figure 3a, CIS exposure resulted in a significant increase in immobility time compared with the control group (*p* < 0.01), while this increase in immobility time observed in the FST was ameliorated by the administration of Xiaoyaosan or rosiglitazone (*p* < 0.01 and *p* < 0.05, respectively).

For the results of the SPF shown in Figure 3b, rats in all groups initially had a similar sucrose preference before stress exposure (baseline condition). However, as shown in Figure 3c, a significant decrease in sucrose preference was measured after 21 days of CIS exposure (*p* < 0.05). Xiaoyaosan or rosiglitazone treatment effectively reversed these changes and significantly increased sucrose preference compared with the CIS group (both *p* < 0.05).

### 2.3. Effects of Xiaoyaosan on Plasma Cholesterol (CHOL), Triglyceride (TG), Low-Density Lipoprotein (LDL-C), High-Density Lipoprotein (HDL-C), Cortisol (CORT) and Insulin Levels in Rats Exposed to CIS

As shown in Table 1, the plasma CHOL, HDL-C, LDL-C and TG levels were significantly lower in rats exposed to stress than in the control group (*p* < 0.001). Both Xiaoyaosan and rosiglitazone treatment increased the CHOL level (*p* < 0.001 and *p* < 0.05, respectively). Additionally, after treatment with Xiaoyaosan, there was a significant increase in the HDL-C level and a decrease in the LDL-C level (*p* < 0.05), but the level of TG did not markedly change (*p* > 0.05). No markable differences in HDL-C, LDL-C and TG levels were observed in the rosiglitazone group (*p* > 0.05).

**Figure 4:**
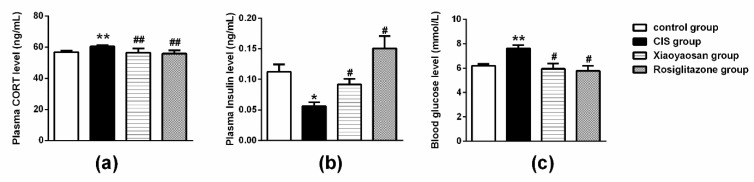
Effects of Xiaoyaosan on plasma CORT, insulin and blood glucose levels in stress-induced rats. (**a**) Plasma CORT levels. (**b**) Plasma insulin levels. (**c**) Blood glucose levels. Data are presented as the means ± SEM from eight rats in each group for determination. * *p* <0.05 or ** *p* < 0.001 versus the control group. ^#^ *p* < 0.05 or ^##^ *p* < 0.001 versus the CIS group.

As shown in Figure 4a, the level of plasma CORT was enhanced when the rats were subjected to 21 days of CIS, which was significantly reversed by the administration of Xiaoyaosan or rosiglitazone (*p* < 0.001).

As shown in Figure 4b, a significant decrease in the level of plasma insulin was observed in all of the stressed rats compared with the level in the control group (*p* < 0.001), whereas the level of plasma insulin in the Xiaoyaosan or rosiglitazone group was noticeably increased (both *p* < 0.05). In addition, the blood glucose level was increased in the CIS-induced rats compared with the control rats (Figure 4c, *p* < 0.001); after treatment with Xiaoyaosan or rosiglitazone, the level of blood glucose was decreased (Figure 4c, both *p* < 0.05).

### 2.4. Effects of Xiaoyaosan on the Expression of SHIP2, p85 and Akt Immunolabelling and mRNAs in the Rat Liver

**Figure 5:**
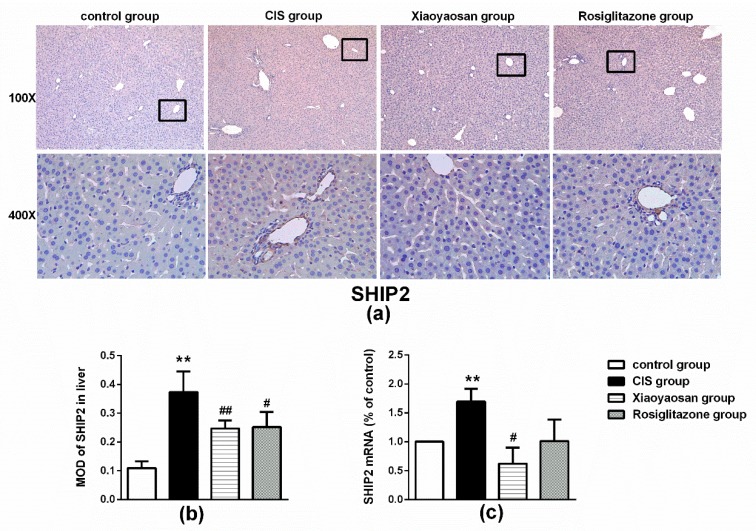
Effects of Xiaoyaosan on the expression of SHIP2 proteins and mRNAs in stress-induced rat liver. (**a**) Representative micrographs of immunohistochemical staining for the SHIP2 proteins (100× and 400× magnification) in the liver. (**b**) Quantitative analysis of SHIP2 protein levels. (**c**) Expression of SHIP2 mRNA in the rat liver. MOD, mean optical density. Data are presented as the means ± SEM from five rats per group. ** *p* < 0.001 versus the control group. ^#^ *p* < 0.05 or ^##^ *p* < 0.001 versus the CIS group.

**Figure 6:**
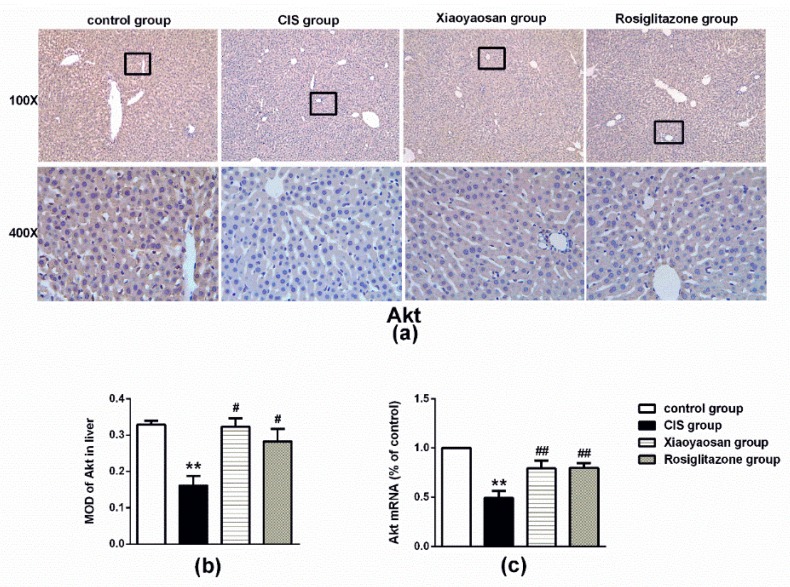

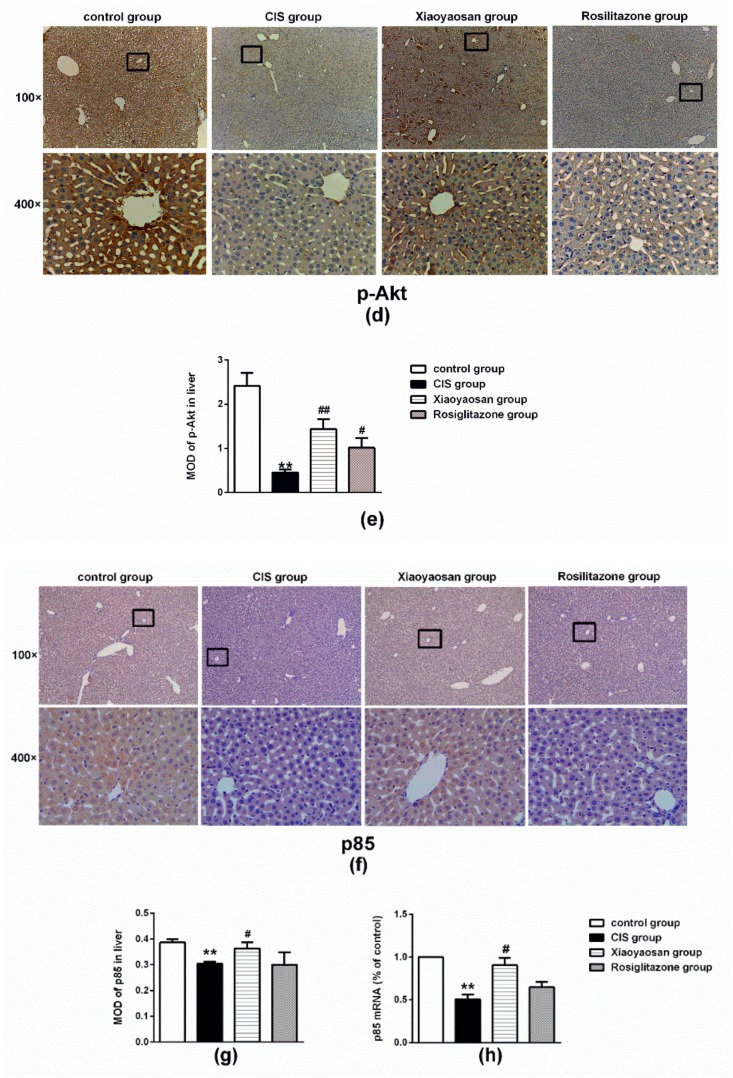
Effects of Xiaoyaosan on the expression of Akt, p-Akt and p85 proteins and mRNAs in a stress-induced rat liver. (**a**) Representative micrographs of immunohistochemical staining for Akt proteins (100× and 400× magnification) in the liver. (**b**) Quantitative analysis of Akt protein levels. (**c**) Expression of Akt mRNA in the rat liver. (**d**) Representative micrographs of immunohistochemical staining for p-Akt protein (100× and 400× magnification) in the liver. (**e**) Quantitative analysis of p-Akt protein levels. (**f**) Representative micrographs of immunohistochemical staining for p85 protein (100×, 400× magnification) in the liver. (**g**) Quantitative analysis of p85 protein levels. (**h**) Expression of p85 mRNA in the rat liver. MOD, mean optical density. Data are presented as the means ± SEM from 5 rats per group for determination. ** *p* < 0.001 versus the control group. ^#^ *p* < 0.05 or ^##^ *p* < 0.001 versus the CIS group.

The level of SHIP2 protein in the liver of rats in the CIS group was markedly higher than that in the control group (Figure 5b, *p* < 0.001), whereas the level in the Xiaoyaosan or rosiglitazone group was significantly reduced (both *p* < 0.001). Furthermore, rats in the CIS group had a higher expression of SHIP2 mRNAs than rats in the control group (Figure 5c, *p* < 0.001), and after Xiaoyaosan treatment, the level of SHIP2 mRNA was decreased (Figure 6c, *p* < 0.05), but there was no significant change in the rosiglitazone group (Figure 5c, *p* > 0.05).

The CIS procedure significantly decreased the expression of Akt protein and mRNA in the liver (Figure 6a–c, *p* < 0.001), while Xiaoyaosan or rosiglitazone treatment significantly increased Akt protein and Akt mRNA expression (*p* < 0.05 and *p* < 0.001, respectively). Moreover, the expression of p-Akt also decreased in the CIS group compared with that of in the control group (Figure 6d,e, *p* < 0.001), after treatment with Xiaoyaosan or rosiglitazone, the reduction was reversed (*p* < 0.001 and *p* < 0.05). Similarly, the stressed rats exhibited significantly lower levels of p85 protein and mRNA than the control rats (Figure 6f–h, *p* < 0.001), and the decrease in the levels of the p85 protein and mRNA was significantly attenuated by Xiaoyaosan treatment (Figure 6g,h, both *p* < 0.05), while the administration of rosiglitazone failed to effectively reverse the lower expression of p85 protein and mRNA (Figure 6g,h, both *p* > 0.05).

## 3. Discussion

In the present study, we investigated the effects of Xiaoyaosan on 21 days of CIS-induced behavioural performance and metabolic responses and explored the potential mechanism underlying the regulatory effects in the liver. Xiaoyaosan significantly improved the body weight and depression-like behaviours of stressed rats. More importantly, exposure to CIS for 21 consecutive days induced a decrease in plasma insulin, CHOL, TG, LDL-C and HDL-C concentrations and an increase in blood glucose. Moreover, the protein and mRNA expression of SHIP2 in the liver was increased, while the protein and mRNA expression of p85 and Akt was decreased. Plasma insulin and blood glucose levels and SHIP2, p85 and Akt protein and mRNA expression levels in the liver were reversed by Xiaoyaosan or rosiglitazone (an insulin sensitizer). Furthermore, Xiaoyaosan effectively regulated the levels of CHOL, LDL-C and HDL-C, but not TG.

Stress is well known to change body weight and food intake in animal models. Several studies have demonstrated that chronic exposure to restraint stress reduces the body weight and food intake of rodents [36,37,38]. Our results showed that restraint stress rapidly induces a marked decrease in body weight that may be due to a reduction in food intake. After treatment with Xiaoyaosan, the body weight of stressed rats significantly increased, but the improvement of food intake in rats was not obvious. The stress response mobilizes the body’s energy stores, and a striking observation was the diversity of metabolic changes that can occur in response to stress [39]. Thus, the effect of Xiaoyaosan on improving rat body weight may be associated with energy metabolism rather than increased food intake.

Increased plasma corticosterone levels are consistent with the physiological response of repeated stress associated with the activation of the HPA axis [40]. Furthermore, as shown in the behavioural test, rats exposed to 21 days of CIS exhibited increased depression-like behaviour, such as significantly increased immobility time within 5 min in the FST and decreased sucrose consumption rate in the SPT. Additionally, these rats also displayed a reduction in the total distance travelled and number of times entering into the centre area in the OFT, indicating deficiency in spontaneous locomotor activity and exploration when the rats were placed in a novel environment. Chronic stress has been shown to increase anxiety and depression-like behaviour in animal models [41,42]. Therefore, these results indicate that chronic restraint stress changes physical and psychological responses. Xiaoyaosan reversed CIS-induced changes, corroborating our previous findings [33], and rosiglitazone showed a similar effect. This may reflect that both Xiaoyaosan and rosiglitazone have some inhibitory effects on rats with chronic stress.

Stress can influence the central nervous system as well as the endocrine, metabolic and immune systems in humans [43], and stress is an important contributor to the amount of blood glucose produced by the liver [44]. Hormonal changes that occur during acute and chronic stress situations can affect glucose homeostasis in both healthy people and in those with diabetes [45]. To determine whether CIS affects glucose homeostasis in the rats, we measured the concentration of plasma insulin and blood glucose in rats that had been exposed to 3 h of restraint immobilization daily for 21 consecutive days. The results showed that there was a significant decrease in plasma insulin with an increase in blood glucose. Therefore, the regulation of chronic stress induced in metabolic parameters appears to go above and beyond the effect of weight alone. A previous study suggested that during the restraint challenge, chronic stress reduced insulin and leptin levels, not just weight restriction [46]. Several studies have also shown reductions in insulin following chronic stress regimens [47,48,49]. The reduction in insulin could have subsequent effects on stress responsiveness since it can modulate HPA axis reactivity [50]. Furthermore, it has been proposed that chronic stress exposure leads to lipid dysregulation, thus altering lipid metabolism [51]. Our study showed that chronic restraint stress reduced plasmatic TC, TG, LDL-C and HDL-C levels. In humans, stress has been associated with increased serum TC levels [52], and stress can increase the risk of cardiovascular disease by altering lipoprotein metabolism [53]. In order to conform to human clinical characteristics much better, several studies use noise, light, temperature and some other stimulation to form chronic stress and use a high-fat diet, and then observe the impact on the lipid metabolism. Chronic stress and a high-fat diet coexist, where the animal’s blood lipid level increased significantly [54,55]. However, the effect of chronic stress alone on lipid metabolism in animals is variable, with an increase or reduction, or even without significant changes [56]. Clinical studies in patients have revealed that most subjects report a preference for palatable food rich in fat and sugar during negative emotions [57]. Therefore, in humans, it is worth considering whether stress exposure directly leads to a rise in serum TC levels or whether a stress-induced palatable food preference and high-fat diet ultimately induce increased blood lipids. In this study, rats were fed a chow diet, and plasma TC, TG, LDL-C and HDL-C levels were reduced significantly after 21 days of CIS, which was consistent with the reduced weight gain and lower food intake. Administration of Xiaoyaosan or rosiglitazone improved plasma insulin and blood glucose levels. In addition, a similar trend towards increased TC, LDL-C and HDL-C levels was also found, while the TG level was virtually unchanged after the Xiaoyaosan treatment. However, rosiglitazone did not improve the levels of TC, TG, LDL-C or HDL-C. Thus, it can be seen that there is a regulatory effect of Xiaoyaosan on both glucose homeostasis and lipid metabolism.

As mentioned in the introduction, SHIP2 is a key regulator of glucose homeostasis and can be targeted when treating diseases that affect insulin metabolism [58]. Transgenic mice expressing catalytically inactive SHIP2 have displayed altered lipid metabolism and insulin secretion [59]. In addition, transgenic mice over-expressing SHIP2 WT are obese and suffer from hepatic insulin resistance [60]. It is well known that insulin stimulation activates the PI3K/Akt pathway, which is responsible for glucose metabolism, including glucose uptake and glycogenesis [61]. SHIP2 specifically hydrolyses PI(3,4,5)P(3) to PI(3,4)P(2) through its 5’phosphatase and negatively regulates PI3-K activity, resulting in a decrease in downstream molecular activity of PI3-K, which negatively regulates the PI3K/Akt-dependent insulin signalling pathway and antagonizes insulin metabolism [62]. Indeed, over-expression of SHIP2 in transgenic mice reduced glucose tolerance and impaired Akt activation in typical insulin target tissues [60].

Interestingly, in agreement with these findings, the current results showed increased expression of SHIP2 protein and mRNA in the rat liver induced by CIS. We also observed the protein and mRNA expression of p85 and Akt in the liver, which could reflect activation of PI3K/Akt signalling. The reduction in p85 and Akt protein and mRNA expression indicated that PI3K/Akt signalling activation was blunted. Notably, these alterations could be effectively restored by treatment with Xiaoyaosan or rosiglitazone. Furthermore, the precise mechanism of action of Xiaoyaosan on insulin metabolism in stressed rats must be further studied. Particularly, alterations in PI3K/Akt signalling activation should be evaluated using SHIP2 inhibitor or SHIP2−/− mice.

## 4. Materials and Methods

### 4.1. Animals

Male Sprague-Dawley rats (SCXK (Jing) 2012-0001), weighing 210 ± 20 g, were purchased from Beijing Vital River Laboratory Animal Technology Limited Company (Beijing, China) and were individually housed in temperature and humidity-controlled (20–24 °C, relative humidity of 30–40%) rooms under a 12 h/12 h light/dark cycle. Rats were allowed to acclimate for at least 1 week before the experiment started. All animal experiments followed guidelines established by the Institutional Animal Care and Use Committee of Beijing University of Chinese Medicine and conformed to the animal welfare guidelines (BUCM-4-2013101501-4001).

The experimental protocols applied in this study were performed in accordance with approved guidelines (Approved number: BUCM-4-2013101501-4001)

### 4.2. Chronic Immobilization Stress (CIS) Procedure

**Figure 7:**
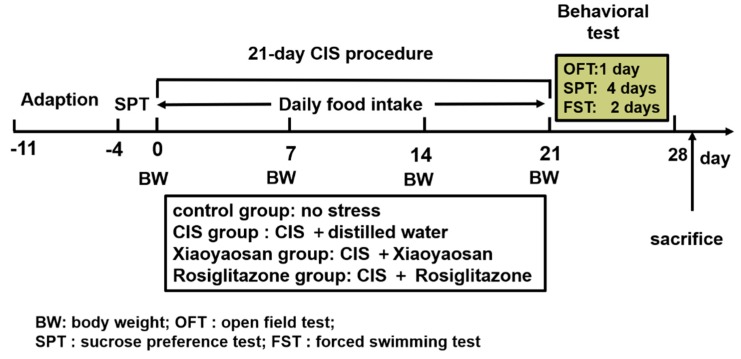
Experimental schedule of this study. After one week of adaption, a sucrose preference test (SPT) was conducted as a baseline to determine the initial behavioural state of the rats. During the 21-day CIS procedure, rats in each group received the respective treatment. The open field test (OFT), SPT and forced swimming test (FST) were conducted to detect depression-like behaviour in rats after CIS stimulation. Animals were sacrificed, and tissue was collected at the end of behavioural testing.

After adaptive feeding, 60 rats were randomly divided into the following 4 groups of 15 rats per group: a control group, a CIS group, a Xiaoyaosan treatment group and a rosiglitazone treatment group. As described in our previous study [19], the CIS rat model was established by using a homemade apparatus composed of wooden T-shaped double-binding platforms. Specifically, the apparatus includes a base platform (20 cm long, 10 cm wide and 2.8 cm thick) and an upper platform (22 cm long and 6.6 cm wide); in front of the upper platform, there is a small frame for fixing the rat head and a groove suitable for placing the limbs. Moreover, there are two adjustable soft adhesive bands surrounding each side of the upper platforms for fixing the rat’s chest and abdomen. The rats in all groups except the control group were immobilized in the apparatus for 3 h every day during dark cycle (19:00 pm–22:00 pm), without access to food or water, for 21 consecutive days from the beginning to the end of the experiment. The experimental schedule is shown in Figure 7.

### 4.3. Drugs and Drug Administration

All of the raw herbs in Xiaoyaosan were purchased from Beijing Tongrentang Group Co., Ltd. (Beijing, China). The quality of the raw herbs was verified by experts from Beijing University of Chinese Medicine School of Pharmacy. The powder was made by Jiuzhitang Co., Ltd (Changsha, China) based on the procedure in the Chinese Pharmacopoeia 2015 Edition [63] and was composed of the following: *Radix Bupleuri* 100 g, *Radix Angelica Sinensis* 100 g, stir-fried *Rhizoma Atractylodis Macrocephalae* 100 g, *Radix Paeoniae Alba* 100 g, *Poria cocos* 100 g, *Radix Glycyrrhizae* 80 g, *Rhizoma Zingiberis Recens* 100 g, and *Herba Menthae* 20 g. Specifically, the main processes used in the production of Xiaoyansan powder included extracting volatile oil from *Radix Bupleuri* 50 g, *Radix Angelica Sinensis* 50 g, *Rhizoma Zingiberis Recens* 100 g and *Herba Menthae* 100 g; drug residue, stir-fried *Rhizoma Atractylodis Macrocephalae*, and *Poria cocos*, etc., were decocted twice with water for 2 h each time, the decoction was combined and filtered, the filtrate was thickened into thick paste, and *Radix Paeoniae Alba* and residual *Rhizoma Atractylodis Macrocephalae* were crushed into fine powder Next, 20 g *Radix Glycyrrhizae* was crushed into fine powder, and the remaining *Radix Glycyrrhizae* was decocted three times with water for 2 h each time. The decoction was then combined and filtered or stored overnight. The supernatant or filtrate was concentrated into a suitable amount, and then, the above thick paste, fine powder, volatile oil and appropriate starch were added to the mix and sealed after drying. The internal batch number of the powder used in this experiment was J2447, and 1 g of the powder contained 2.10 g of raw drug. The molecular structures of the ingredients in Xiaoyaosan were measured by high-performance liquid chromatography-mass spectrometry (LC-MS/MS) (AB SCIEX, Framingham, MA, USA) in our previous study [64].

The Xiaoyaosan powder was dissolved in distilled water and administered via gavage at a dose of 1.845 g/kg/day, 0.1 mL/kg bodyweight (according to the average adult body weight and a dose conversion of 60 kg/day). Rosiglitazone (Jiangsu the Yellow River Pharmaceutical Limited by Share Ltd, 160102, Jiangsu, China) was dissolved in distilled water and administered via gavage at a dose of 3 mg/kg/day, 0.1 mL/kg bodyweight. Rats in the control and CIS groups were given the same volume of distilled water. The distilled water or drugs were intragastrically administered for 21 days.

### 4.4. Body Weight and Food Intake

To investigate the effect of Xiaoyaosan on the physical condition of CIS-induced rats, the body weight and food intake of each rat were evaluated. The body weight was recorded every week until the end of the experiment, and starting from the first day of modelling, food intake was recorded every day from 22:00 pm to 10:00 am. The body weight and food intake within the same week (1st, 2nd, and 3rd weeks) were compared among the four groups.

### 4.5. Behavioural Testing

Open field test (OFT): Locomotor activity and exploratory behaviour were measured using the OFT. The OFT was performed in a wooden square black box (125 × 125 × 50 cm) with a central zone (75 × 75 cm), and a video camera installed 2.5 m above the apparatus to record animal movements. Animals were placed individually in the box for 5 min and in a sound-proof observation room illuminated with controlled light. The number of central and peripheral squares entered, the time spent in central area, the distance travelled, and the animal’s velocity were analysed using Observer 5.0 software (Noldus, Netherlands) and EthoVision 3.0 software (Noldus, Netherlands).

Sucrose preference test (SPT): Sensitivity to reward can be assessed using the SPT. The animals were given access to water with and without different concentrations of sucrose, and the preference rate for sucrose was analysed. This test is often used to assess the level of depression [65]. The SPT was performed at one-week intervals during the stress period (starting from baseline) and lasted for 4 days. All rats were allowed free access to two bottles containing 1% sucrose solution for the first 24 h. Then, all rats were allowed to choose between two bottles, one containing tap water and the other containing 1% sucrose solution for 24 h. To avoid bottle side preference, the sides (left and right) of the two bottles in the housing were switched after 12 h. On the third day, rats were deprived of food and water for 24 h. After 72 h, rats were exposed for 1 h to two identical bottles, one filled with 1% sucrose solution and the other filled with water. Sucrose preference was determined as the ratio of the consumed sucrose solution relative to the total water consumption during the 1-h test.

Forced swimming test (FST): The FST is the most extensive trial used to evaluate the effect of antidepressants [66]. Antidepressants reduce immobility, which is used as a major predictor of antidepressant activity [67]. As previously described [68], the FST procedure includes the following standard protocol: a 15-min pretest swim and a 5-min test swim 24 h later. Briefly, rats were forced to swim individually for 15 min on the final day of administration in a clear glass cylinder (height, 24 cm; diameter, 19 cm), which was filled to 20 cm of water at room temperature (23 ± 1 °C). After 24 h, the rats were forced to swim again for 5 min. The duration of immobility was scored during the 5 min test period. Each rat was recorded as being immobile based on the activity of the four limbs and the ratio of body area that was under or above the water surface.

### 4.6. Enzyme-Linked Immunosorbent Assay (ELISA)

The serum CORT and insulin levels were determined with commercial ELISA kits (CORT, Enzo ADI-900-097, New York, NY, USA; Insulin, 80-INSRT-E01, E10, New Hanpshire, NH, USA). According to the manufacturer’s instructions, 100 μL of each standard or sample was added to 96-well plates coated with primary antibodies followed by incubation at 37 °C for 1 h. The wells were washed with 0.05% Tween 20 (PBST). After incubation and several washes, 100 μL of 1:1000 horseradish peroxidase (HRP)-conjugated secondary antibody diluted in PBST was added to each well, and the plates were incubated at 37 °C for 1 h. Then, all wells were washed with PBST three times and incubated with 100 μL per well of TMB (3,3’,5,5’-tetramethylbenzidine solution) substrate until a yellow colour appeared. The addition of 2 mol/L H2SO4 stopped the reaction. Sample absorbance at 450 nm was read using a Multiskan™ GO (Thermo Fisher Scientific, Waltham, MA, USA) detector system.

Plasma CHOL, HDL-C, LDL-C, TG and blood glucose were determined by using an automatic biochemical analyser (CX4/Pro, Beckman, Brea, CA, USA). The test was carried out by the Biochemical Laboratory of Scientific Research Center of Beijing University of Traditional Chinese Medicine.

### 4.7. Quantitative Real-Time Polymerase Chain Reaction (qRT-PCR)

The expression of p58, Akt and SHIP2 mRNA in the liver was detected by qRT-PCR. Total RNA was isolated from the rat livers with Trizol reagent following the manufacturer’s manual (Applied Biosystems, Waltham, MA, USA). The concentration of total RNA was determined by a spectrophotometer (Eppendorf, Germany), and the purity of RNA was measured using 1% agarose gel electrophoresis. The RNA from each sample was used to synthesize first-strand cDNA using a RevertAid First Strand cDNA Synthesis Kit (Thermo Fisher Scientific, Waltham, MA, USA) on a C1000 TouchTM Thermal Cycler (Bio-Rad, California, CA, USA). Primers were designed based on published mRNA sequences and synthesized by Sangon Biotech Co., Ltd (Shanghai, China). The sequences for the primers were as follows: GAPDH, 5′-CCATTCTTCCACCTTTGAT-3′ (Forward) and 5′-TGGTCCAGGGTTTCTTACT-3′ (Reverse); p85, 5′-GCCTCAGTGGACTTGGATGTGTTC-3′ (Forward) and 5′-GTCTTCGGAGCTTGGTACTTCTTGG-3′ (Reverse); Akt, 5′-CTCAACAACTTCTCAGTGGCACAATG-3′ (Forward) and 5′-GCAGGCAGCGGATGATGAAGG-3′ (Reverse); SHIP2, 5′-TTTGCAGCAGCAGAGCCTAC-3′ (Forward) and 5′-GTGCTGTGGAGCTCATGTCC-3′ (Reverse). qRT-PCR was performed on a CFX96 Real-time PCR System (Bio-Rad, CA, USA) with Power SYBR® Green PCR Master Mix (Thermo Fisher Scientific, Waltham, MA, USA) in a final volume of 20 μL with the following thermal cycling conditions: 95 °C for 10 min, 40 cycles of 95 °C for 15 s and 55 °C for 1 min, followed by 65 °C for 5 s and 95 °C for 15 s. The mRNA of these genes was quantified using the comparative threshold cycle (2^−ΔΔCt^) method. All samples were tested in triplicate. Target gene expression was compared with the housekeeping gene GAPDH. After PCR, the melting curve was obtained and analysed.

### 4.8. Liver Tissue Immunohistochemical Staining

Immunohistochemical staining was performed on the paraffin-embedded liver biopsies in accordance with conventional procedures previously described [46], which briefly included antigen retrieval, inactivation of endogenous peroxidase, blocking nonspecific antigens and reaction with antigen antibodies. Slices were incubated with primary antibodies (p85, Proteintech, 1:100; Akt, Proteintech, 1:100; SHIP2, Abcam, 1:50) overnight at 4 °C. Afterwards, slides were incubated with the corresponding secondary antibodies (anti-mouse, ZSGB-BIO, Beijing, China, diluted 1:2,000; and anti-rabbit, ZSGB-BIO, Beijing, China, 1:2,000) for 2 h at room temperature. Then, sections were placed in a DAB (diaminobezidin 3,3) reagent (Invitrogen, Carlsbad, CA, USA) for 5–10 min at room temperature. After further rinsing in 0.1 mol PBS, sections were re-stained with haematoxylin and mounted on gelatin-coated slides for observation. Images of positive staining were captured at 400× magnification using a light microscope (BX53, Olympus Co., Tokyo, Japan). Image-Pro Plus 6.0 software (Media Cybernetics, Bethesda, MD, USA) was used to obtain the MOD.

### 4.9. Statistical Analysis

All data were analysed using SPSS 20.0 software (Chicago, IL, USA) and expressed as the means ± standard deviation (SD). All statistical analyses were performed with a one-way analysis of variance (ANOVA) followed by Fisher’s least significant difference (LSD) test for multiple comparisons when equal variances were assumed. Homogeneity of variances was not satisfied, and thus, one-way ANOVA with Welch’s robust test of equality of means with post hoc Dunnett’s T3 test were used to analyse significant differences among groups. ANOVA with repeated measures was used to compare body weight and food intake. A *p*-value < 0.05 was considered statistically significant.

## 5. Conclusions

The present study demonstrates that CIS induces a reduction in body weight and food intake and leads to a depression-like phenotype. Moreover, reductions in plasma insulin and lipid levels, which may be associated with increased SHIP2 expression in the liver, were observed. Xiaoyaosan, a classic traditional Chinese herbal medicine, reverses the decreases in body weight and plasma insulin level, restores hepatic SHIP2 expression and attenuates the behavioural consequences of CIS. These findings not only provide evidence for the effects of Xiaoyaosan on metabolic abnormalities caused by chronic stress, which may be related to regulating SHIP2 expression to improve PI3K/Akt signalling activity in the liver, but also shed new light on the clinical use of Xiaoyaosan.

## Figures and Tables

**Table 1:** Effects of Xiaoyaosan on plasma cholesterol (CHOL), triglyceride (TG), low-density lipoprotein (LDL-C) and high-density lipoprotein (HDL-C) in stress-induced rats.

Group	CHOL (mmol/L)	HDL-C (mmol/L)	LDL-C (mmol/L)	TG (mmol/L)
Control group	3.551 ± 0.508	1.642 ± 0.146	1.092 ± 0.085	0.552 ± 0.121
CIS group	2.243 ± 0.443 **	1.115 ± 0.053 **	0.652 ± 0.160 **	0.352 ± 0.065 **
Xiaoyaosan group	2.755 ± 0.410 ^##^	1.322 ± 0.214 ^#^	0.508 ± 0.100 ^#^	0.395 ± 0.056
Rosiglitazone group	2.710 ± 0.466 ^#^	1.190 ± 0.186	0.677 ± 0.116	0.413 ± 0.076

Notes: Data are presented as the means ± SD from six rats in each group for determination. ** *p* < 0.001 versus the control group. ^#^ *p* < 0.05 or ^##^ *p* < 0.001 versus the CIS group.
